# A specific synbiotic-containing amino acid-based formula in dietary management of cow’s milk allergy: a randomized controlled trial

**DOI:** 10.1186/s13601-019-0241-3

**Published:** 2019-01-15

**Authors:** Adam T. Fox, Harm Wopereis, Marleen T. J. Van Ampting, Manon M. Oude Nijhuis, Assad M. Butt, Diego G. Peroni, Yvan Vandenplas, David C. A. Candy, Neil Shah, Christina E. West, Johan Garssen, Lucien F. Harthoorn, Jan Knol, Louise J. Michaelis, ASSIGN study group

**Affiliations:** 1 Guy’s and St Thomas’ Hospitals NHS Foundation Trust, London, UK; 2 Danone Nutricia Research, Nutricia Advanced Medical Nutrition, Uppsalalaan 12, 3584 CT Utrecht, The Netherlands; 3 Wageningen University, Wageningen, The Netherlands; 4 Royal Alexandra Children’s Hospital, Brighton, UK; 5 University Hospital Verona, Verona, Italy; 6 UZ Brussel, Vrije Universiteit Brussel, Brussels, Belgium; 7 Great Ormond Street, London, UK; 8 Umeå University, Umeå, Sweden; 9 Utrecht Institute for Pharmaceutical Sciences, Utrecht University, Utrecht, The Netherlands; 10 Great North Children’s Hospital, Newcastle upon Tyne, UK

**Keywords:** Bifidobacterium breve M-16V, Gut microbiota, Prebiotic, Probiotic, Cow’s milk allergy, Symptoms

## Abstract

**Background:**

Here we report follow-up data from a double-blind, randomized, controlled multicenter trial, which investigated fecal microbiota changes with a new amino acid-based formula (AAF) including synbiotics in infants with non-immunoglobulin E (IgE)-mediated cow’s milk allergy (CMA).

**Methods:**

Subjects were randomized to receive test product (AAF including fructo-oligosaccharides and *Bifidobacterium breve* M-16V) or control product (AAF) for 8 weeks, after which infants could continue study product until 26 weeks. Fecal percentages of bifidobacteria and *Eubacterium rectale*/*Clostridium coccoides* group (*ER/CC*) were assessed at 0, 8, 12, and 26 weeks. Additional endpoints included stool markers of gut immune status, clinical symptoms, and safety assessments including adverse events and medication use.

**Results:**

The trial included 35 test subjects, 36 controls, and 51 in the healthy reference group. Study product was continued by 86% and 92% of test and control subjects between week 8–12, and by 71% and 80%, respectively until week 26. At week 26 median percentages of bifidobacteria were significantly higher in test than control [47.0% vs. 11.8% (*p* < 0.001)], whereas percentages of *ER/CC* were significantly lower [(13.7% vs. 23.6% (*p* = 0.003)]. Safety parameters were similar between groups. Interestingly use of dermatological medication and reported ear infections were lower in test versus control, *p* = 0.019 and 0.011, respectively. Baseline clinical symptoms and stool markers were mild (but persistent) and low, respectively. Symptoms reduced towards lowest score in both groups.

**Conclusion:**

Beneficial effects of this AAF including specific synbiotics on microbiota composition were observed over 26 weeks, and shown suitable for dietary management of infants with non-IgE-mediated CMA.

*Trial Registration* NTR3979

**Electronic supplementary material:**

The online version of this article (10.1186/s13601-019-0241-3) contains supplementary material, which is available to authorized users.

## Introduction

Cow’s milk allergy (CMA) is a common childhood condition [1], but optimal management can be affected by challenges in obtaining an accurate diagnosis [2–5]. These challenges are greatest in infants with non-immunoglobulin E (IgE) CMA [6] who account, in certain regions, for one-quarter of confirmed CMA cases [1]. Gastrointestinal and skin symptoms characterize non-IgE-mediated CMA, and symptoms can present from severe to the most commonly presented moderate to mild symptoms [7, 8]. Few clinical studies have been published on effective management of patient populations with non-IgE-mediated CMA [9] because of difficulty in diagnosis and lack of validated tests [1, 7, 10, 11].

Research into the pathogenesis of childhood allergies and associated aberrant gut microbiota composition have shown a possible role for early-life gut microbiota in immune-system development [12–15]. The beneficial effects of breastfeeding on gut microbiota and immune maturation in early life [16, 17] provided a scientific rationale for investigations into prebiotics and probiotics in infants requiring formula [18–24].

Amino acid-based formula (AAF) is recommended for severe or complex CMA or when extensively hydrolyzed formula (eHF) fails to resolve symptoms [3]. Clinical studies have confirmed the safety of AAF containing synbiotics (prebiotics and probiotics) in infants [25, 26]. Based on these studies, a randomized controlled trial (ASSIGN) investigated an AAF containing specific synbiotics in infants with non-IgE-mediated CMA. Previously published primary outcome, which was at week 8 timepoint, showed that 8 weeks use of the AAF including specific synbiotics positively modified fecal microbiota by increasing bifidobacteria, who are typically abundant in healthy breast fed infants [27], and reducing *Eubacterium rectale*/*Clostridium coccoides* group (*ER/CC)*, typically more abundant in the more adult phase of microbiota development [28], compared with AAF alone, resulting in levels approximating those observed in a healthy breast-fed reference group [29]. This paper now reports the full 26 weeks study results on fecal microbiota composition, safety, and explored markers for gut health and immune status.

## Methods

ASSIGN was a double-blind, randomized controlled multicenter trial, with a separate non-randomized healthy, breastfed reference group (Netherlands Trial Resister NTR3979). The trial was approved by the ethics committees of participating centers and all parents/guardians provided written informed consent. Detailed methods including inclusion and exclusion criteria, sample size determination, randomization protocol and blinding, study assessments, and the primary outcome measure, have been published previously [29].

**Fig. 1:**
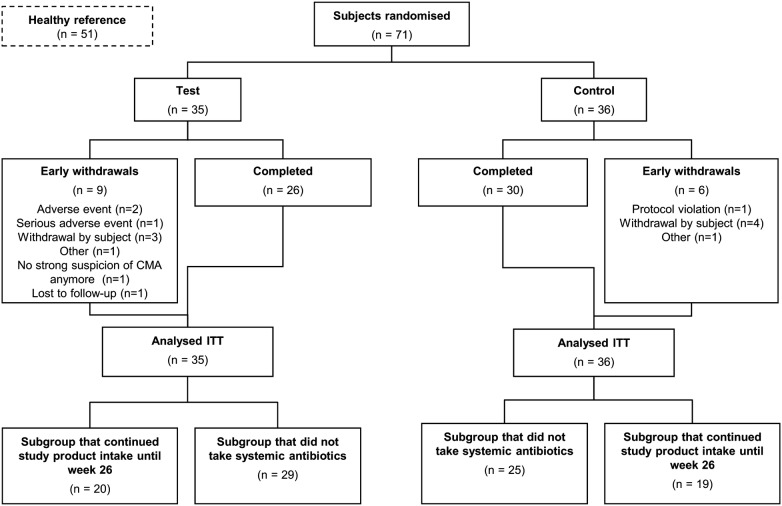
CONSORT diagram showing the flow of subjects in the randomized arms. *ITT* intention to treat. Early withdrawal-related adverse events were constipation (n = 1) and infantile colic (n = 1) and a related serious adverse event (n = 1) was viral laryngitis. The events were reported as unlikely and not related to study formula

In brief, we enrolled subjects < 13 months old, presenting with persistent symptoms, with a strong suspicion of non-IgE-mediated CMA who were randomized to receive test or control formula for 8 weeks. Clinical history or strong suspicion of an allergic reaction to cow’s milk protein was based on a robust diagnostic work-up, collectively designed by a multidisciplinary team of clinicians, comprising pediatric gastroenterology, allergy, and immunology specialists. The defined inclusion criteria were as published [29] and included a negative specific IgE test (ImmunoCAP), and/or a negative skin prick test with cow’s milk protein, if a test was performed (testing was not mandatory per protocol). In addition at study entry the subjects had at least one of the following (GI) symptoms related to inclusion of cow’s milk protein in their diet: faltering growth; frequent regurgitation or vomiting; extended periods of diarrhea with a negative stool examination (negative microbiology and virology laboratory tests); soft stool constipation; blood in stool; iron-deficiency anemia due to occult or macroscopic blood loss in stools not due to infection or dietary insufficiency; endoscopically confirmed eosinophilic enteropathy; or persistent distress or colic (> 3 h per day at least 3 days per week over 3-week period). Infants were excluded for the following reasons: birth weight < 2500 g, < 37 weeks gestation requiring specific premature infant formula at study entry, severe concurrent illness, functional GI symptoms without suspicion of atopy and food allergy, immune, autoimmune or gluten sensitive enteropathy, food protein-induced enterocolitis syndrome, acute or chronic diarrhea secondary to a confirmed infectious gastroenteritis, behavioral disorders with food aversion or food phobia, GI surgery, syndromes commonly associated with functional GI disorders, and the use of probiotics, systemic antibiotics or anti-mycotic drugs 4 weeks preceding study entry. Two weeks after randomization symptom resolution was evaluated and subjects with persistent symptoms were reassessed by the investigator and only subjects with suspicion of, or confirmed, non-IgE CMA continued in the study. Subjects not eligible at reassessment were withdrawn (Fig. 1). Subjects in the healthy, breastfed reference group were age matched to week 8 of the randomized groups. The test formula, a hypoallergenic, nutritionally complete AAF (Neocate LCP; Nutricia Advanced Medical Nutrition, Liverpool, UK) contained a prebiotic blend of chicory-derived neutral oligofructose, long-chain inulin (BENEO-Orafti SA, Oreye, Belgium) (9:1 ratio at a total concentration of 0.63 g/100 ml) and a probiotic strain *Bifidobacterium breve* M-16V (Morinaga Milk Industry, Tokyo, Japan) at a concentration of 1.47 × 10^9^ colony-forming units/100 mL formula. The control formula was a commercially available AAF (Neocate LCP; Nutricia Advanced Medical Nutrition, Liverpool, UK). After 8 weeks, subjects received a prescribed formula appropriate for their condition and age per clinicians’ choice and practice. If subjects were prescribed an AAF they continued with their randomly assigned formula.

Stool samples were collected at week 0, 8, 12, and 26, as reported previously [29]. Percentages of bifidobacteria and *Eubacterium rectale/Clostridium coccoides* group (*ER/CC*), *Clostridium histolyticum*, and *Clostridium lituseburense* groups were analyzed by fluorescent in situ hybridization (FISH), as described previously [30].

To explore the potential of a number of fecal markers in non-IgE-mediated food allergy (FA) this study assessed secretory immunoglobulin A (sIgA) [31], eosinophil cationic protein (ECP), calprotectin (FC) [32], and alpha1-antitrypsin in feces.

Under clinical supervision, parents/guardians recorded clinical symptoms before starting study formula and over 3 days during weeks 1, 4, 8, 12, and 26; symptom diaries were reviewed by the investigator during clinic visits. Parent-reported rating scales for skin, respiratory, gastrointestinal, and general symptoms, and clinician-reported skin symptoms via SCORAD were determined as described previously [29]. Briefly, parent reported scores were collected using a four-point scale where a score of (1) was taken as normal without symptoms. Additional stool records, study formula intake, and diet evaluation were collected as described previously.

The frequency and severity of adverse events, allergic symptoms, stool characteristics, use of concomitant medications, and standard anthropometric measurements were used to assess safety and tolerability over 26 weeks.

Safety analyses used the all-subjects treated (AST) dataset and all other analyses were performed on the intention-to-treat dataset (ITT), defined as all randomized subjects. The primary endpoint (percentage of bifidobacteria and *ER/CC* at week 8) in the randomized groups and the healthy breastfed reference group were reported previously [29]. Exploratory outcomes included allergic symptoms, fecal markers, and bacterial groups which were statistically tested for differences between the treatment groups by using ANCOVA or van Elteren depending on normality of the residuals. Growth parameters were compared using ANCOVA and concomitant medications using Fisher’s exact test. Stool consistency was assessed using ANCOVA. Subgroup analyses were done on all randomized subjects who did not take systemic antibiotics during the study period to week 26 and on those who did continue study product intake to week 26. The missing data in the outcome parameters were considered as Missing At Random (MAR). For the parameters subject to limit of detection (LOD), the following rule was applied: If a value is below detection limit and the percentage of values below detection limit is at most 30%, then the value was replaced with LOD/2. Statistical analyses were performed by using SAS^®^ (SAS Enterprise Guide version 4.3 or higher) for Windows (SAS Institute Inc., Cary, NC). Results are expressed as mean values ± SD unless stated otherwise.

## Results

Subject characteristics were well balanced between groups and have been reported previously [29]. Figure 1 summarizes the flow of 71 subjects with non-IgE CMA in the randomized groups from week 0 to week 26, and shows that 26/35 (74.3%) in the test group and 30/36 (83.3%) in the control group completed the study to week 26. At baseline 35/71 subjects (49.3%) were being fed with an AAF, 32.4% with hydrolysate, 15.5% with whole protein formula, while 2.8% were breastfed. The majority ITT population (20/35 test and 19/36 control) continued with their assigned study formula until week 26 as per their clinical recommendation (Additional file 1: Table S1). In the final study period, week 12 to 26, 5 subjects in the test group and 2 subjects in the control group received cow’s milk formula (Additional file 1: Table S1).

During the study in both groups the most common reason for early termination was withdrawal of the subject (Fig. 1; 8.6% and 11.1% in test and control, respectively). Overall 9 subjects in test group reported as withdrawal reason: AE (n = 2), sAE (n = 1), withdrawal by subject (n = 3), no strong suspicion of CMA at 2 week evaluation (n = 1), other (1), lost to follow up (1). In the control group (n = 6) reported reasons for withdrawal were: protocol violation (n = 1), withdrawal by subject (n = 4), and other (n = 1). Early withdrawal-related adverse events were constipation (n = 1) and infantile colic (n = 1) and a related serious adverse event (n = 1) was viral laryngitis. The events were reported as unlikely and not related to study formula. Early withdrawal rates were not different between groups (Fig. 1).

### Fecal microbiota

**Fig. 2:**
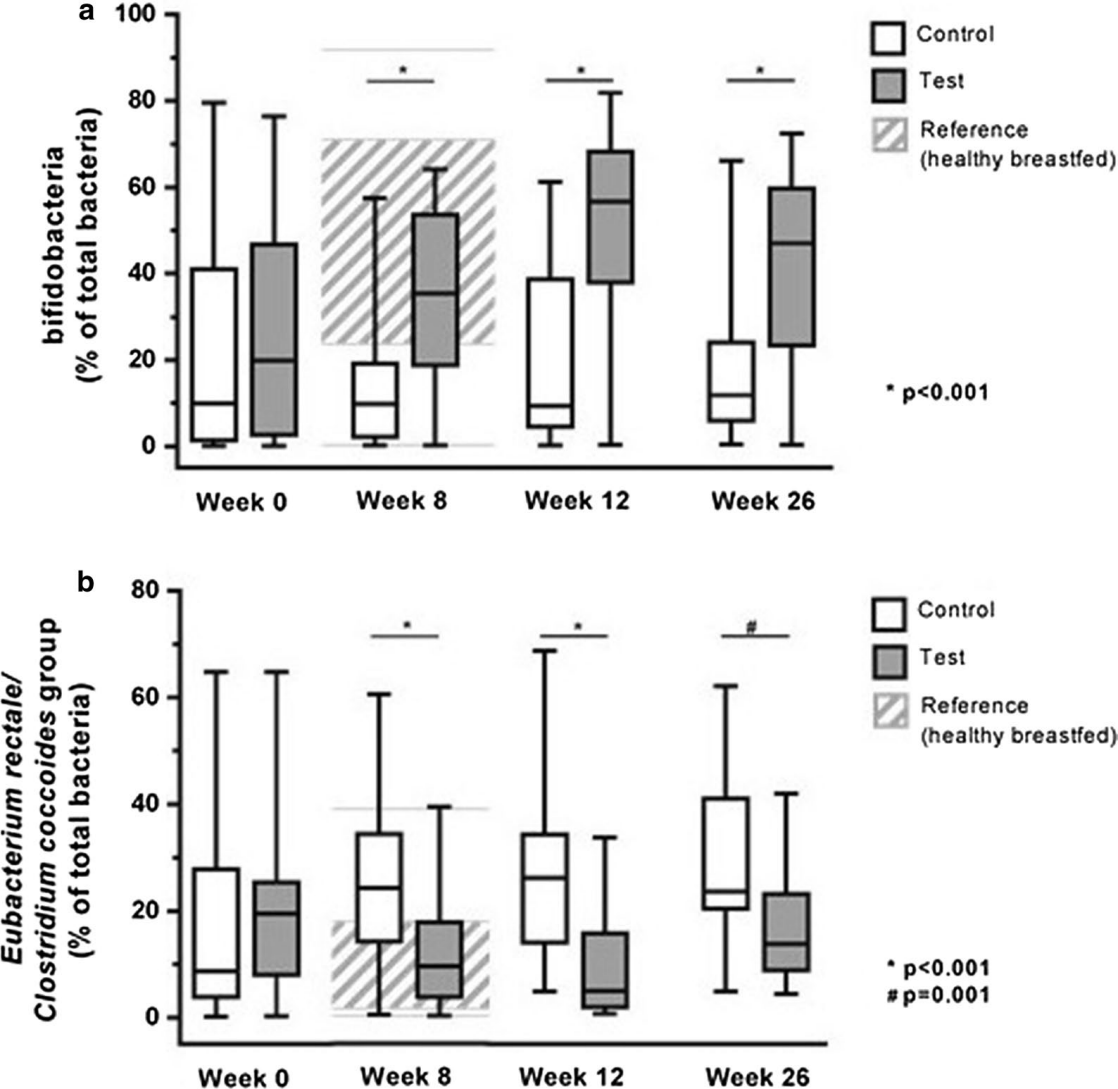
Percentages of bifidobacteria (**a**) and ER/CC (**b**) at weeks 0 to 26 in ITT. The grey shaded area represents the sample 25th to 75th percentile of the healthy reference group (healthy, breastfed subjects—age matched to CMA subjects at week 8) and the grey horizontal lines represent the minimum and maximum values of this healthy reference group. The bottom and top edges of the box are located at the sample 25th and 75th percentiles. The center horizontal line is drawn at the 50th percentile (median). The whiskers of the box plots show the minimum and maximum values. *P* values are based on ANCOVA comparing test versus control with week 8, 12 or 26 values as outcome, stratification factor (skin or gastrointestinal symptoms) and imputed baseline values as covariate and intervention as fixed effect

The between-group differences in microbiota composition seen at week 8 (primary trial endpoint) were maintained with longer study follow-up. At weeks 12 and 26, the test group had a higher percentage of bifidobacteria and a lower percentage of *ER/CC* compared with the control group (Fig. 2). At week 26 statistically significant effects on fecal microbiota were maintained in subgroup analyses comprising 54 subjects (29 test and 25 control) who did not take systemic antibiotics during the study period to week 26 (Table 1a), and in subgroup analyses of 39 subjects (20 test and 19 control) who continued taking their allocated study product until week 26 (Table 1b). The complementary subgroup of subjects receiving antibiotics during the study period also showed higher mean percentages of bifidobacteria and lower *ER/CC* in test versus control; however, the number of subjects in these complementary groups were too small for statistical interpretation (6 vs. 11, respectively; data not shown). Complementary subgroup of subjects that did not continue with study product till week 26 showed similar trends but were also too small to draw any conclusions (9 vs. 10 subjects; data not shown).

**Table 1:** Percentages of bifidobacteria and *ER/CC* at weeks 8, 12 and 26 in: (a) subgroup of subjects that did not take systemic antibiotics during the study period; (b) subgroup of subjects that received study product until week 26

(A)					
Bacteria (%)	Week	Test (N = 29)	Control (N = 25)	*P* value^a^	HBR (N = 51)
Bifidobacteria, mean [95% CI]	0	28.30	20.75	0.267^b^	
		[18.7–37.9]	[10.8–30.7]		
	8	36.31	14.48	0.001	48.08
		[26.6–46.0]	[6.9–22.1]		[40.6–55.5]
	12	53.09	17.61	0.003	
		[40.3–65.9]	[6.3–29.0]		
	26	43.28	14.07	< 0.001	
		[31.5–55.0]	[7.0–21.2]		
*ER/CC* cluster, mean [95% CI]	0	18.48	16.73	0.705^b^	
		[12.3–24.7][	[9.5–24.0]		
	8	13.44	24.99	0.014	10.38
		[8.1–18.8]	[17.7–32.2]		[7.4–13.4]
	12	10.16	34.12	< 0.001	
		[4.2–16.1]	[25.0–43.3]		
	26	15.92	31.72	0.002	
		[10.6–21.2]	[23.5–40]		

(B)					
Bacteria (%)	Week	Test (N = 20)	Control (N = 19)	*P* value^a^	HBR (N = 51)
Bifidobacteria, mean [95% CI]	0	26.63	17.74	0.231^b^	
		[14.8–38.4]	[7.9–27.5]		
	8	38.40	13.06	0.002	48.08
		[23.4–53.4]	[5.9–20.3]		[40.6–55.5]
	12	49.74	17.09	0.002	
		[28.0–71.5]	[6.4–27.8]		
	26	48.77	15.12	< 0.001	
		[37.0–60.6]	[7.0–23.3]		
*ER/CC* cluster, mean [95% CI]	0	23.55	13.42	0.074^b^	
		[15.1–32.0]	[5.5–21.3]		
	8	6.31	25.52	< 0.001	10.38
		[3.7–9.0]	[16.8–34.2]		[7.4–13.4]
	12	7.45	32.11	0.002	
		[2.1–12.7]	[22.5–41.7]		
	26	13.07	33.92	< 0.001	
		[8.2–17.9]	[24.8–43.0]		

*HBR* healthy, breastfed reference group (infants within a similar age range to subjects in the randomized groups at study week 8 [29]). *N* is number of subjects, *CI* confidence interval

^a^Based on ANCOVA comparing Test versus Control with stratification factor and baseline value as covariate and treatment as fixed effect

^b^Interference based on t-test

The percentage of fecal *Clostridium histolyticum* group decreased from week 0 to week 8 in the test group (median change − 0.5; Q1–Q3: − 2.4 to 0.1), but increased in the control group (median change 0.4; Q1–Q3: 0.1–0.8). The changes from week 0 were statistically significantly different between test and control groups at weeks 8 (*P* = 0.002), 12 (*P* = 0.002), and 26 (*P* < 0.001). There were no differences between groups in levels of *Clostridium lituseburense* group at any time point.

### Exploratory markers in stools

At week 8 fecal sIgA, FC, and alpha1-antitrypsin were all within the range of healthy breastfed reference. Median fecal ECP levels were below 25th percentile of the healthy breastfed reference. At each time point treatment groups were not statistically significantly different (Additional file 2: Fig. S1).

### Stool characteristics

Stool characteristics were not statistically significantly different between test and control groups at weeks 0, 8, 12, and 26 (data not shown).

### Clinical symptoms

Overall, clinical symptoms were mild (but persistent) at baseline (Additional file 3: Fig. S2). During the study mean scores for vomiting, spitting up, and gas wind, reduced in both groups towards the lowest possible score (1 = none) and were not statistically significantly different between groups (Additional file 3: Fig. S2c). Respiratory related symptoms reduced over time (coughing, blocked nose), or remained similar to level at study entry (wheezing, close to 1 at study entry).

Crying (due to irritability), visual signs of discomfort (*e.g.* back arching), and skin symptoms reduced towards the lowest possible score in both groups at wk 26 (Fig. 2a, c).

Baseline median [Q1–Q3] SCORAD was 6.0 [0.0–19.0] and 9.0 [0.0–20.0] in test and control group, respectively. Median scores decreased between week 0 and 26 by 6.0 (Q1–Q3: − 13.0, 0.0) and 7.0 (Q1–Q3: − 13.0, 0.0) in the test and control groups, respectively (data not shown).

### Growth and safety parameters

Measured growth parameters were all within the expected ranges for age (Additional file 4: Table S2). Head circumference (between group difference 0.57; 95% CI: 0.13, 1.02) and head circumference-for-age Z-score (between group difference 0.41; 95% CI: 0.07, 0.75) were statistically significantly different between test and control groups at week 8 (*P* = 0.013 and *P* = 0.019, respectively), but not at weeks 12 and 26. Weight, length, weight gain, length gain, weight-for-age Z-score, length-for-age Z-score and weight-for-length Z-score were not statistically significantly different between test and control groups at any time points.

Concomitant medications were consistent with the studied population of young children. Use of dermatological medications was statistically significantly lower in test than control group at week 26 (Table 2). Specific subcategories ‘emollients and protectives’ (*p* = 0.023) and ‘antifungal agents’ (*p* = 0.054) were lower in the test group (Table 2).

**Table 2:** Concomitant medication use (number of subjects taken medication) in All subjects treated (AST)

	Test (N = 35)	Control (N = 35)	*P*-value (Fisher’s exact test)
Concomitant medication [N (%)]			
Overall			
Any concomitant medication	25 (71.4%)	29 (82.9%)	0.394
Subcategory^*^			
Dermatologicals	6 (17.1%)	16 (45.7%)	0.019
Antibiotics and chemotherapeutics	1 (2.9%)	2 (5.7%)	1.00
Antifungals	0 (0%)	5 (14.3%)	0.054
Antipruritics^a^	0 (0%)	2 (5.7%)	0.493
Antiseptics and disinfectants	1 (2.9%)	4 (11.4%)	0.356
Corticosteroids, dermatological preparations	6 (17.1%)	9 (25.7%)	0.561
Emollients and protectives	2 (5.7%)	10 (28.6%)	0.023

*Only categories (of total 10 categories) with a statistically significant difference (*P* < 0.05) are shown

^a^Antipruritics, including antihistamines and anesthetics

The types and number of adverse events were well balanced between the groups. Adverse events were recorded in 25/35 (71%) subjects in the test group and 28/35 (80%) subjects in the control group (Additional file 5: Table S3). The most commonly reported adverse events during the 26-week study period were gastrointestinal disorders (including constipation, diarrhea, flatulence, and gastro-esophageal reflux disease) and infections and infestations (nasopharyngitis, upper respiratory tract infection, and ear infection). Although the overall incidence of adverse events was not different between groups, the incidence of infections and infestations subcategory ‘ear infections’ was significantly lower in the test group than in the control group (0% vs. 20%, respectively; *P* = 0.011). Serious adverse events were reported in 5 subjects during the study to week 26 (Additional file 5: Table S3), and all were categorized as not related or unlikely to be related to study product. The types of reported (serious) adverse events (gastroesophageal reflux disease, laryngitis viral, and bronchiolitis that required hospitalization (n = 2), and an anaphylactic reaction to pineapple) and their severity were consistent with the studied population of young children with CMA.

## Discussion

The primary aim of this study was to investigate the fecal microbiota and analysis demonstrated that 8 weeks use of test product significantly (*p* < 0.001) increased bifidobacteria and decreased *ER/CC* [29] with percentages close to levels seen in age-matched healthy breastfed subjects [29]. We now report that the differences in fecal microbiota between groups were maintained for the full study period until 26 weeks, clinical symptoms reduce in both groups towards lowest possible score, and the formula including oligofructose, long-chain inulin, and *B. breve* M-16V was well tolerated and suitable for management of CMA symptoms.

Dietary management of CMA include eHF for mild cases and AAF for more severe or complex cases, or when eHF fails to resolve symptoms [3]. While these approaches are recommended in guidelines, they do not address the gut microbiota, which is now widely recognized to play an important role in immune development [13, 33, 34]. The synbiotic composition of the test product was developed following preclinical and clinical research showing positive effects on microbiota and potential management of allergy [35–39]. Administration of these specific synbiotics with AAF resulted in significant changes in fecal microbiota composition, which were maintained, as reported in this study, at week 26 in the ITT population. Significant differences between test and control groups were maintained at all time points in both the ITT population and a subgroup analysis of subjects who continued test product for 26 weeks. These observations suggest that the AAF including synbiotics sustains changes in gut microbiota composition, as measured in feces. Several other factors can affect the development and diversity of the gut microbiota in infancy, including exposure to systemic antibiotics [40]. A subgroup analysis of changes in fecal microbiota in subjects who did not receive any systemic antibiotics during the 26-week study period showed that AAF including synbiotics increased bifidobacteria and decreased *ER/CC* at all time points. The complementary subgroup receiving antibiotics during the study, showed similar results. Although the number of subjects was too small for statistical interpretation, it may suggest that the effects on gut microbiota by AAF including synbiotics can even be maintained in a CMA population receiving systemic antibiotics.

Interestingly the current full 26-week data shows there was significantly lower use of agents for dermatological purposes and a lower incidence of ear infections in the test group compared with the control group, suggesting possible systemic effects of synbiotics beyond modification of gut microbiota. The prevalence of eczema at baseline was well balanced between groups. While less frequent use of dermatologic medications in the test group suggests the possibility of an improvement in skin symptoms, no difference was detected by parent-assessed reporting. Interpretation of these data may be confounded by the heterogeneity of pre-study feeding regimens and differences in clinical practice between centers; further study is needed to confirm a potential effect of this specific synbiotics-containing AAF on skin symptoms. Clinical studies of prebiotics and probiotics have shown some improvement or reduction in eczema in infants with allergic conditions [30, 41–43], but none of these studies specifically included subjects with non-IgE CMA. The effect of improving gut microbiota composition on overall gut health and immune status remains to be determined. Clinical studies have shown that AAF or eHF containing prebiotics and/or probiotics have positive effects on microbiota in allergic infants [19–26, 41–43], however, heterogeneity in study populations and differences in the formulas studied, and the pre-and/or probiotics they contain, mean that it is difficult to compare these studies.

Fecal markers can non-invasively give an insight into the ‘innate’ immune status of the gut mucosa and a few have been identified as markers to diagnose gastrointestinal conditions, such as Crohn’s disease [44]. In contrast, data regarding this type of markers related to FA, especially in non-IgE-mediated FA, remain scarce and controversial. Research suggests sIgA plays a role in mucosal immune defense, whereas ECP and FC may reflect mucosal levels of eosinophils and neutrophils, respectively [45–47]. Fecal alpha1-antitrypsin has been suggested as a marker of protein-losing enteropathy [48]. In the present study, these fecal markers were within healthy reference ranges. However, mean fecal ECP was lower than the 25th percentile of the health breastfed reference. This is in line with previously reported association of elevated fecal ECP and breast feeding [49]. Although fecal calprotectin has been suggested as potential a marker to monitor response to exclusion diets, or challenge proven FA [50], this only has been confirmed in children of 1 year or older. Studies indicate that levels of FC are depending on age [51] and diagnostic accuracy may be difficult to interpret in infants, which we can confirm based on current observations. To our knowledge these markers have never been reported in this specific non-IgE CMA population and in FA infants at this age. The results indicate these markers to be inconclusive in this study population. It is to be investigated whether this is due to the relatively mild to moderate nature of clinical symptoms, or different (immune) markers would be more suitable to investigate mechanisms involved in non-IgE mediated FA.

Likely due to use of hypoallergenic formula at baseline and associated relatively low symptoms at baseline, our analysis did not show detectable differences in clinician-assessed or parent-reported clinical symptoms between groups. Hypoallergenic formula use prior to study enrolment limits the ability of the study to investigate differences between groups in symptom scores. Nevertheless, the study was not designed to explore improvements in clinical symptoms and immune parameters and indicates that a larger study with earlier randomization is required to study clinical effects in this non-IgE CMA population.

This study also confirmed that AAF including synbiotics was well tolerated and no safety concerns were revealed with longer follow-up. The incidence and severity of adverse events at week 26 were not significantly different between test and control groups suggesting that administration of AAF with synbiotics for at least 8 weeks, and up to 26 weeks, is well tolerated and associated with growth and development within the normal range. Previous studies in infants with CMA also found no safety concerns with the addition of *B. breve* M-16V and prebiotics to AAF [25, 26].

While seeking to expand the body of evidence for synbiotics in subjects with non-IgE CMA, our study is inherently limited by the challenges in making and confirming a specific and accurate diagnosis. An allergen challenge was not mandatory to confirm diagnosis, potentially allowing subjects with other than strict CMA allergic presentations to be in the trial population. We developed a robust diagnostic work-up [29] to mitigate this possibility and inclusion required careful symptom assessment and specific IgE testing and skin-prick testing (if assessed) to exclude any IgE-mediated CMA. Overall caution must be taken in interpreting results, particularly in the case of subgroup analyses with smaller numbers of subjects.

These results are specific to the test product containing a unique combination of prebiotics and *B. breve* M-16V and cannot be extrapolated to other AAFs or different synbiotic formulations.

In conclusion, use of the AAF including specific synbiotics investigated in this study resulted in a sustained improvement in gut microbiota composition over 26 weeks. Clinical symptoms reduced in both groups towards lowest possible score. The AAF with the specific synbiotics is safe and suitable for dietary management of infants with suspected non-IgE-mediated CMA.

## Additional files

**Additional file 1: Table** **S1.** Summary of formula used after study week 8 by subjects that completed the study till study week 12 and 26, respectively.

**Additional file 2: Figure S1.** Box plot of exploratory markers in stools: (a) fecal sIgA, (b) ECP, (c) calprotectin, and (d) alpha-1-antitrypsin. The grey area represents the sample 25th to 75th percentile of the healthy subjects and the grey lines represent the minimum and maximum values of the healthy subjects (matched on age at Week 8 only). Horizontal line in box plot is the 50th percentile (median), whiskers of the box plots show the minimum and maximum values. The diamonds represent the mean values.

**Additional file 3: Figure S2.** Parent-reported, clinician-evaluated, symptoms at weeks 0, 4, 8, 12, and 26 assessed on a 4-point rating scale specific for each symptom, with score 1 as lowest possible score. **(a)** *Skin symptoms* (redness, oozing, crusting, itchiness, dryness, and nappy rash) were rated as 1: none, 2: slight, 3: some, 4: a lot. **(b)** *Respiratory symptoms blocked nose and wheezing* rated as 1: none, 2: mild, 3: moderate, 4: severe, and *coughing* was rated as 1: none, 2: 1-2 times/day, 3: 3-5 times/day, 4: more than 5 times/day. **(c)** G*eneral and gastrointestinal symptom vomiting* were rated as 1: none, 2: 1–2 times/day, 3: 3–4 days/day, 4: more than 4 times/day; *spitting*-*up* as 1: none, 2: after some feeds, 3: after all feeds, 4: between and after feeds; *gas/wind* as 1: none; 2: slight; 3: some; 4: a lot; *sleep pattern last night* as 1: normal, 2: awake once, 3: awake 2–3 times, 4: awake more than 3 times; ease of settling or burping after feeds as 1: no problem at all, 2: slight difficulty, 3: some difficulty, 4: very difficult; *visual signs of discomfort (e.g. back arching)* as 1: none, 2: slight, 3: some, 4: a lot; and *crying (due to irritability)* as 1: none, 2: up to 1 h, 3: 1–3 h, 4: more than 3 h. Data are shown as mean values ± 95% confidence interval limits.

**Additional file 4: Table** **S2.** Descriptive summary of growth parameters at weeks 0, 8, 12, and 26.

**Additional file 5: Table** **S3.** Adverse events in test and control groups (AST) from first study intake until the end of the study (week 26).

## Abbreviations

AAF: amino acid-based formula
AST: all-subjects treated
*B. breve* M-16V: *Bifidobacterium breve* M-16V
CMA: cow’s milk allergy
ECP: eosinophil cationic protein
eHF: extensively hydrolyzed formula
*ER/CC*: *Eubacterium rectale/Clostridium coccoides* group
FC: fecal calprotectin
FISH: fluorescent in situ hybridization
IgE: immunoglobulin E
ITT: intention-to-treat
LOD: limit of detection
sIgA: secretory immunoglobulin A
