# Supplementary material for: A specific synbiotic-containing amino acid-based formula in dietary management of cow’s milk allergy: a randomized controlled trial
Source: Clin Transl Allergy. 2019 Jan 15;9:5. doi: 10.1186/s13601-019-0241-3 (PMC6332540; doi:10.1186/s13601-019-0241-3)
Supplement: Supplementary file 1 — Additional file 1: Table S1. Summary of formula used after study week 8 by subjects that completed the study till study week 12 and 26, respectively. [file 13601_2019_241_MOESM1_ESM.docx]

**Supplementary Digital Content**

**Supplementary Table 1.** Summary of formula used after study week 8 by subjects that completed the study till study week 12 and 26, respectively.

|  | | **Test, n (%)** | **Control, n (%)** |
| --- | --- | --- | --- |
| **Formula used until week 12** | Study product | 24 (86%) | 24 (92%) |
|  | Cow’s milk* | 2 (7%) | 1 (4%) |
|  | Soy | 2 (7%) | 1 (4%) |
| **Formula used until week 26** | Study product | 20 (71%) | 19 (80%) |
|  | Cow’s milk* | 5 (18%) | 2 (8%) |
|  | Soy | 2 (7%) | 3 (13%) |
|  | Hydrolysate | 1 (4%) | 0 (0%) |

**Subjects consuming cow’s milk or a combination of cow’s milk and soy formula were included in the cow’s milk group.*
