# Supplementary material for: A specific synbiotic-containing amino acid-based formula in dietary management of cow’s milk allergy: a randomized controlled trial
Source: Clin Transl Allergy. 2019 Jan 15;9:5. doi: 10.1186/s13601-019-0241-3 (PMC6332540; doi:10.1186/s13601-019-0241-3)
Supplement: Supplementary file 2 — Additional file 2: Figure S1. Box plot of exploratory markers in stools: (a) fecal sIgA, (b) ECP, (c) calprotectin, and (d) alpha-1-antitrypsin. The grey area represents the sample 25th to 75th percentile of the healthy subjects and the grey lines represent the minimum and maximum values of the healthy subjects (matched on age at Week 8 only). Horizontal line in box plot is the 50th percentile (median), whiskers of the box plots show the minimum and maximum values. The diamonds represent the mean values. [file 13601_2019_241_MOESM2_ESM.docx]

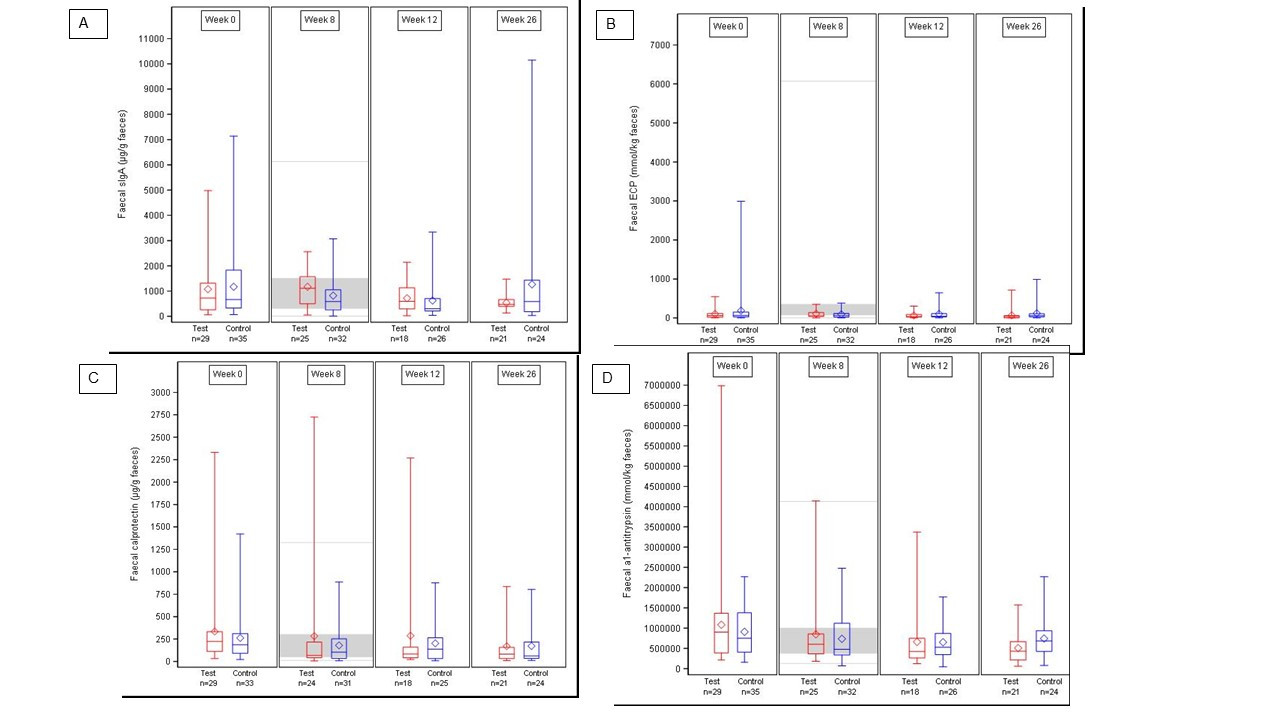
**Supplemental Figure S1. Box plot of exploratory markers in stools: (a) fecal sIgA, (b) ECP, (c) calprotectin, and (d) alpha-1-antitrypsin.** The grey area represents the sample 25th to 75th percentile of the healthy subjects and the grey lines represent the minimum and maximum values of the healthy subjects (matched on age at Week 8 only). Horizontal line in box plot is the 50th percentile (median), whiskers of the box plots show the minimum and maximum values. The diamonds represent the mean values.
