# Supplementary material for: A specific synbiotic-containing amino acid-based formula in dietary management of cow’s milk allergy: a randomized controlled trial
Source: Clin Transl Allergy. 2019 Jan 15;9:5. doi: 10.1186/s13601-019-0241-3 (PMC6332540; doi:10.1186/s13601-019-0241-3)
Supplement: Supplementary file 3 — Additional file 3: Figure S2. Parent-reported, clinician-evaluated, symptoms at weeks 0, 4, 8, 12, and 26 assessed on a 4-point rating scale specific for each symptom, with score 1 as lowest possible score. (a) Skin symptoms (redness, oozing, crusting, itchiness, dryness, and nappy rash) were rated as 1: none, 2: slight, 3: some, 4: a lot. (b) Respiratory symptoms blocked nose and wheezing rated as 1: none, 2: mild, 3: moderate, 4: severe, and coughing was rated as 1: none, 2: 1-2 times/day, 3: 3-5 times/day, 4: more than 5 times/day. (c) General and gastrointestinal symptom vomiting were rated as 1: none, 2: 1–2 times/day, 3: 3–4 days/day, 4: more than 4 times/day; spitting-up as 1: none, 2: after some feeds, 3: after all feeds, 4: between and after feeds; gas/wind as 1: none; 2: slight; 3: some; 4: a lot; sleep pattern last night as 1: normal, 2: awake once, 3: awake 2–3 times, 4: awake more than 3 times; ease of settling or burping after feeds as 1: no problem at all, 2: slight difficulty, 3: some difficulty, 4: very difficult; visual signs of discomfort (e.g. back arching) as 1: none, 2: slight, 3: some, 4: a lot; and crying (due to irritability) as 1: none, 2: up to 1 h, 3: 1–3 h, 4: more than 3 h. Data are shown as mean values ± 95% confidence interval limits. [file 13601_2019_241_MOESM3_ESM.docx]

**
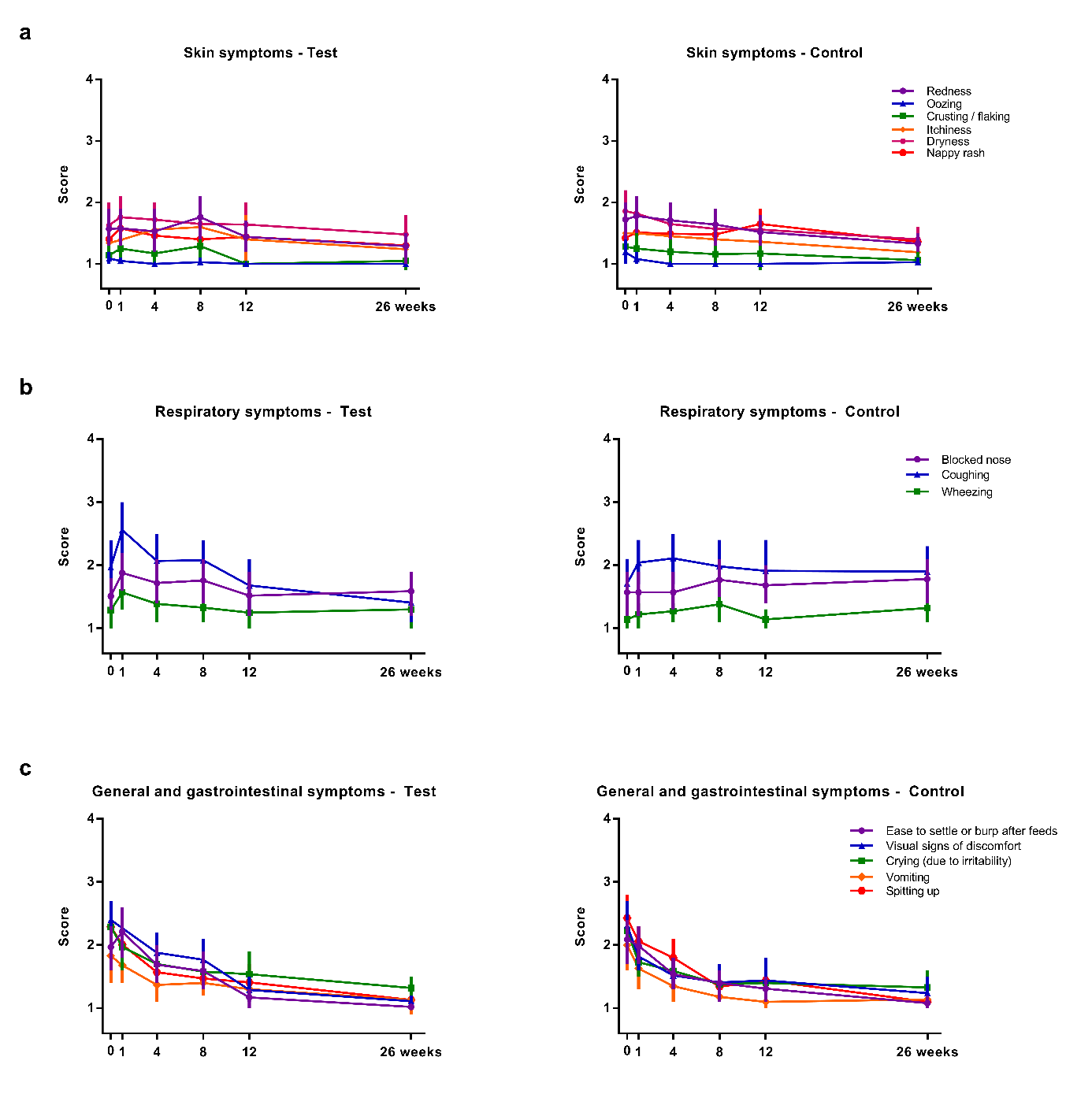
Supplemental Figure S2.** Parent-reported, clinician-evaluated, symptoms at weeks 0, 4, 8, 12, and 26 assessed on a 4-point rating scale specific for each symptom, with score 1 as lowest possible score. **(a)***Skin symptoms* (redness, oozing, crusting, itchiness, dryness, and nappy rash) were rated as 1: none, 2: slight, 3: some, 4: a lot. **(b)** *Respiratory symptoms* *blocked nose and wheezing* rated as 1: none, 2: mild, 3: moderate, 4: severe, and *coughing* was rated as 1: none, 2: 1-2 times/day, 3: 3-5 times/day, 4: more than 5 times/day. **(c)** G*eneral and gastrointestinal symptom* *vomiting* were rated as 1: none, 2: 1-2 times/day, 3: 3-4 days/day, 4: more than 4 times/day; *spitting-up* as 1: none, 2: after some feeds, 3: after all feeds, 4: between and after feeds; *gas / wind* as 1: none; 2: slight; 3: some; 4: a lot; *sleep pattern last night* as 1: normal, 2: awake once, 3: awake 2-3 times, 4: awake more than 3 times; ease of settling or burping after feeds as 1: no problem at all, 2: slight difficulty, 3: some difficulty, 4: very difficult; *visual signs of discomfort (e.g. back arching)* as 1: none, 2: slight, 3: some, 4: a lot; and *crying (due to irritability)* as 1: none, 2: up to 1 hour, 3: 1-3 hours, 4: more than 3 hours. Data are shown as mean values +/- 95% confidence interval limits.
