# Supplementary material for: A specific synbiotic-containing amino acid-based formula in dietary management of cow’s milk allergy: a randomized controlled trial
Source: Clin Transl Allergy. 2019 Jan 15;9:5. doi: 10.1186/s13601-019-0241-3 (PMC6332540; doi:10.1186/s13601-019-0241-3)
Supplement: Supplementary file 4 — Additional file 4: Table S2. Descriptive summary of growth parameters at weeks 0, 8, 12, and 26. [file 13601_2019_241_MOESM4_ESM.docx]

**Supplementary Table 2.** Descriptive summary of growth parameters at weeks 0, 8, 12, and 26.

|  | **Statistic** | **Test (N=35)** | | | |  | ***Control (N=35)*** | | | |
| --- | --- | --- | --- | --- | --- | --- | --- | --- | --- | --- |
|  |  | Week 0 | Week 8 | Week 12 | Week 26 |  | Week 0 | Week 8 | Week 12 | Week 26 |
| Weight-for-age Z score | Mean  ± SD | −0.213 ±1.122 | 0.104 ±0.877 | 0.220 ±0.957 | 0.347 ±0.930 |  | −0.315 ±0.895 | −0.137 ±0.871 | −0.071 ±0.726 | 0.185 ±0.789 |
| Length-for-age Z score | Mean  ± SD | 0.307 ±1.486 | 0.570 ±1.178 | 0.661 ±1.117 | 0.328 ±1.084 |  | 0.269 ±0.951 | 0.322 ±0.783 | 0.446 ±0.766 | 0.411 ±0.924 |
| Weight-for-length Z score | Mean  ± SD | −0.499 ±1.132 | −0.178 ±0.840 | −0.085 ±0.962 | 0.322 ±0.797 |  | −0.573 ±1.200 | −0.356 ±1.011 | −0.349 ±0.816 | 0.053 ±0.954 |
| Head circumference-for-age Z score | Mean  ± SD | 0.488 ±1.184 | 0.954 ±0.948 | 0.927 ±0.953 | 0.872 ±1.128 |  | 0.430 ±1.042 | 0.495 ±1.120 | 0.672 ±0.958 | 0.683 ±0.924 |

N is number of subjects. SD = standard deviation.
