# Supplementary material for: A specific synbiotic-containing amino acid-based formula in dietary management of cow’s milk allergy: a randomized controlled trial
Source: Clin Transl Allergy. 2019 Jan 15;9:5. doi: 10.1186/s13601-019-0241-3 (PMC6332540; doi:10.1186/s13601-019-0241-3)
Supplement: Supplementary file 5 — Additional file 5: Table S3. Adverse events in test and control groups (AST) from first study intake until the end of the study (week 26). [file 13601_2019_241_MOESM5_ESM.docx]

**Supplementary Table 3.** Adverse events in test and control groups (AST) from first study intake until the end of the study (week 26).

|  | | ***Test***  ***(N=35)*** | ***Control***  ***(N=35)*** | ***P-value (Fisher’s exact test)*** |
| --- | --- | --- | --- | --- |
| ***Adverse events, N (%)*** | |  |  |  |
| Overall | Any adverse event | 25 (71.4%) | 28 (80%) | 0.578 |
|  | Serious adverse events* | 3 (8.6%) | 2 (5.7%) |  |
| Severity | Mild | 18 (51.4%) | 16 (45.7%) |  |
|  | Moderate | 6 (17.1%) | 10 (28.6%) |  |
|  | Severe | 1 (2.9%) | 2 (5.7%) |  |
| Preferred term description^#^ | Gastrointestinal disorders | 15 (42.9%) | 17 (48.6%) | 0.811 |
|  | Infections and infestations | 15 (42.9%) | 22 (62.9%) | 0.150 |
| *Subcategory* | *Ear infections* | 0 (0%) | 7 (20.0%) | 0.011 |

*3 subjects reported 3 serious adverse events (gastroesophageal reflux disease, laryngitis viral, and bronchiolitis that required hospitalization and medication) in the test group and 2 subjects reported 2 serious adverse events (bronchiolitis that required hospitalization and an anaphylactic reaction to pineapple). All serious adverse events were considered not related, or unlikely to be related, to the study product.

^#^The two most frequent reported preferred terms of adverse event are shown. Only the significantly different subcategory, of total 25 subcategories, within infections and infestations is shown.
